# Mechanisms of receptor tyrosine kinase activation in cancer

**DOI:** 10.1186/s12943-018-0782-4

**Published:** 2018-02-19

**Authors:** Zhenfang Du, Christine M. Lovly

**Affiliations:** 1 Department of Medicine, Division of Hematology and Oncology, Vanderbilt University Medical Center, Nashville, TN 37232 USA; 2 Vanderbilt-Ingram Cancer Center, Vanderbilt University Medical Center, Nashville, TN 37232 USA

**Keywords:** Receptor, Tyrosine kinase, Cancer, Mutation, Chromosomal rearrangement, Targeted therapy, Tyrosine kinase inhibitor (TKI), Oncogene

## Abstract

Receptor tyrosine kinases (RTKs) play an important role in a variety of cellular processes including growth, motility, differentiation, and metabolism. As such, dysregulation of RTK signaling leads to an assortment of human diseases, most notably, cancers. Recent large-scale genomic studies have revealed the presence of various alterations in the genes encoding RTKs such as *EGFR*, *HER2*/*ErbB2*, and *MET*, amongst many others. Abnormal RTK activation in human cancers is mediated by four principal mechanisms: gain-of-function mutations, genomic amplification, chromosomal rearrangements, and / or autocrine activation. In this manuscript, we review the processes whereby RTKs are activated under normal physiological conditions and discuss several mechanisms whereby RTKs can be aberrantly activated in human cancers. Understanding of these mechanisms has important implications for selection of anti-cancer therapies.

## Background

Receptor tyrosine kinases (RTKs) are a subclass of tyrosine kinases that are involved in mediating cell-to-cell communication and controlling a wide range of complex biological functions, including cell growth, motility, differentiation, and metabolism. There are 58 known RTKs in humans [1, 2], and all RTKs share a similar protein structure comprised of an extracellular ligand binding domain, a single transmembrane helix, and an intracellular region that contains a juxtamembrane regulatory region, a tyrosine kinase domain (TKD) and a carboxyl (C-) terminal tail [3]. Dysregulation of RTK signaling leads to many human diseases, especially cancer. Given the advent of the genomic era and the implementation of next generation sequencing (NGS) in cancer research as well as routine clinical practice, mutational landscapes have been established in almost all types of human tumors [4]. These genomic studies have revealed the presence of several different types of alterations in the genes encoding RTKs such as *EGFR*, *HER2*/*ErbB2*, *MET*, amongst many others. The presence of recurrent RTK genomic alterations raises the question about how they function in cancer development and how to best treat cancer patients whose tumors harbor certain RTK mutations. In this manuscript, we review the processes whereby RTKs are activated under normal physiological conditions and discuss several mechanisms whereby RTKs can be aberrantly activated in human cancers, which have important implications for selection of anti-cancer therapies.

## Mechanisms of RTK activation under normal physiologic conditions

**Fig. 1:**
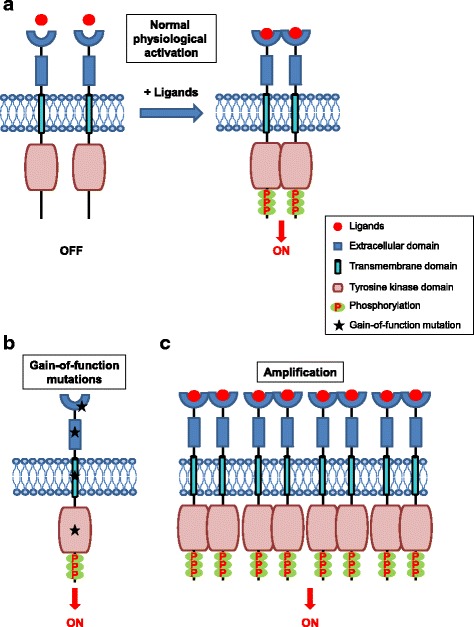
Mechanisms of physiological and oncogenic RTK activation. **a** Schematic representation of RTK activation in normal physiology. RTKs are activated through formation of inter-molecular dimerization in the presence of ligands, resulting in kinase activation and phosphorylation of the receptor C-terminal tail. **b** Schematic representation of potential gain-of-function mutations in the various subdomains of an RTK. The mutations lead to constitutive activation of the RTK, typically in the absence of ligand. **c** Overexpression of RTKs – often as a result of genomic amplification of the RTK gene - leads to increased local concentration of receptors

RTKs are generally activated by receptor-specific ligands. Growth factor ligands bind to extracellular regions of RTKs, and the receptor is activated by ligand-induced receptor dimerization and/or oligomerization [5] (Fig. 1a). For most RTKs, the resultant conformational changes enable *trans*-autophosphorylation of each TKD and release of the *cis*-autoinhibition [6]. This conformational change allows the TKD to assume an active conformation. Autophosphorylation of RTKs also recruits and activates a wide variety of downstream signaling proteins which contain Src homology-2 (SH2) or phosphotyrosine-binding (PTB) domains. These domains bind to specific phosphotyrosine residues within the receptor and engage downstream mediators that propagate critical cellular signaling pathways [7].

### Ligand-induced dimerization of RTK extracellular regions

In general, there are four modes of RTK dimerization which lead to activation of the tyrosine kinase domain. In the first mode, receptor dimerization is completely ligand mediated without any direct contact between the extracellular regions of the two receptors, such as in the case of TrkA (NGF receptor) [8]. In the second mode, dimerization is instead completely receptor mediated without any physical interaction between two activating ligands, as in the case of ErbB family members (EGFR, HER2/ErbB2, HER3/ErbB3, and HER4/ErbB4) [9]. In the third mode, ligand homodimers bind to two receptor molecules, which then interact with each other across the dimer interface, such as the case for KIT (SCF receptor) [10]. In the fourth mode, in addition to a combination of bivalent ligand binding and direct receptor-receptor contacts, accessory molecules also participate in receptor dimerization. For example, the FGFR family of RTKs uses heparin or heparan sulfate as accessory molecules in this mode [11, 12].

Notably, a subset of RTKs forms dimers or high-order oligomers even without activating ligands. The receptors stay in dynamic equilibrium between monomers and dimers. For EGFR and many other RTKs, monomers predominate before ligand-binding [13]. For the insulin receptor (IR), dimers predominate even without ligands [14, 15]. The pre-formed dimers exist as either “inactive” or “active” form. The “inactive” dimers are likely in dynamic equilibrium with “active” dimers. An active dimer will be stabilized by ligand binding, whereas an inactive dimer will be activated by ligand binding through conformational changes. In both scenarios, the ligand binding will shift the equilibrium to the formation of ligand-induced dimerization [13–15].

The ErbB family is of particular interest in cancer biology, and therefore discussed here in additional detail. The extracellular regions of the ErbB receptors family include four subdomains (I-IV) [16]. In the absence of ligands, the intracellular TKD is inactive, and the extracellular region adopts a “tethered” configuration in which the dimerization arm (a β-hairpin within subdomain II of the ECD) is entirely buried by intra-molecular interactions with domain IV and forms intra-molecular autoinhibitory interactions. Ligand simultaneously binds to two sites (subdomain I and subdomain III) within the extracellular region of one receptor, rather than spanning two separate receptors as seen for NGF [8], SCF [10], or FGF receptor [17]. Ligand binding induces a dramatic conformational change that “extends” the extracellular region and exposes the previously buried dimerization arm to an active conformation. With the dimerization arm exposed, the extracellular region of the receptor dimerizes [18], inducing intracellular conformational changes so that they can enable kinase activation [9].

### Activation of intracellular tyrosine kinase domains

Numerous studies have been performed to determine how physiological information is transmitted from the exterior to the interior of the cells. Before activation, the TKD is in a condition of *cis*-autoinhibition by certain intra-molecular interactions unique for each receptor [19, 20]. Ligand-induced dimerization releases this *cis*-autoinhibition. FGFR, IR, and IGF-1R receptors are autoinhibited by the activation loop, which directly contacts the active site of the kinase and disrupts ATP and substrate binding [21, 22]. KIT and Eph receptors are regulated by juxtamembrane autoinhibition, in which the juxtamembrane region interacts with components within the active site of the kinase—thereby stabilizing an inactive state [20, 23]. For the TEK, MET, and RON (MST-1R) receptors, the C-terminal tail contacts the active site of the TKD, thus inhibiting substrate access [19]. This interaction stabilizes an inactive conformation which exerts a strong autoinhibition on kinase activity. Ligand-induced dimerization induces *trans*-phosphorylation of key tyrosine residues, resulting in destabilization of these autoinhibitory interactions and therefore, allowing the kinase to assume an active conformation.

Again, calling out the unique properties of the ErbB family of RTKs – the kinase activity of these receptors is activated through a unique allosteric mechanism whereby the C-lobe of one kinase domain in the dimer pair (the so called ‘activator’ kinase) physically contacts the N-lobe of the other kinase domain in the dimer pair (the so called ‘receiver’ kinase). This physical interaction induces conformational changes in the N-lobe of receiver kinase [9] which induces activation of the ‘receiver’ kinase domain and *trans*-phosphorylation of tyrosine residues in the C-terminal tail of the ‘activator’. Phosphorylation of the activation loop is not involved in this mechanism [24, 25].

### Mechanism of activation of downstream signaling

Activation and subsequent autophosphorylation of RTKs results in the recruitment of a wide range of downstream signaling proteins. Most autophosphorylation sites function as binding sites for SH2 or PTB domain containing signaling proteins. SH2 domain containing proteins can be recruited directly to the receptor, or indirectly to the receptor through docking proteins which bind to RTKs via their PTB domains. Docking proteins function as “assembly platforms” to recruit additional signaling molecules containing SH2 or other domains [5, 26]. The presence of several phosphotyrosines and the involvement of various docking proteins confer activated RTKs the ability to recruit and regulate a wide range of signaling pathways including RAS/MAPK, PI-3 K/AKT, and JAK2/STAT signaling. Therefore, RTKs function as a node which transfers complicated information regarding cell growth and migration from the extracellular milieu ultimately to the cell nucleus to activate transcriptional pathways involved in regulating many cellular processes.

### Summary of RTK activation under normal physiologic conditions

Several decades of intricate structural and biochemical studies have revealed the complicated mechanisms whereby RTKs are activated in a ligand mediated way to propagate cellular signals. A detailed understanding of receptor physiology is crucial to fully understand how and why oncogenic mutations in RTKs disrupt this normal biology, resulting in a dysregulation of cell growth, aberrant cell signaling, and altered metabolism in tumor cells.

## Oncogenic activation of receptor tyrosine kinases

Under normal physiologic conditions, the level of RTK activity is tightly balanced by the mechanisms described above and by additional molecules, including tyrosine phosphatases [27]. RTKs acquire transforming abilities through several mechanisms, and the final consequence is the disruption of the balance between cell growth/proliferation and cell death [5]. When temporal and spatial regulation are taken into consideration, dysregulated RTK signaling becomes even more complicated [28]. Constitutive activation may confer oncogenic properties upon normal cells and trigger RTK-induced oncogenesis [29]. Four principal mechanisms lead to constitutive RTK activation in human cancers: gain-of-function mutations, genomic amplification, chromosomal rearrangements, and / or autocrine activation [6]. Here, we discuss these four oncogenic activating mechanisms including a special intragenic duplication – kinase domain duplication (KDD).

### Activation by gain-of-function mutations

A gain-of-function mutation in an RTK leads to aberrant downstream signal transduction, not subjected to the normal ‘checks and balances’ that occur with physiological signaling. Of particular interest is the identification and functional characterization of ‘driver mutations’ - defined as mutations that can confer a selective growth advantage to the cells [4]. These ‘driver mutations’ can shed light on the understanding of cancer initiation and progression and can also provide potential opportunities for targeted treatments. Somatic mutations in the genes encoding RTKs typically cluster in evolutionally conserved residues, such as the DFG motif in the kinase activation loop and around the nucleotide-binding pocket. These conserved residues (D, F, and G) play key roles in ATP binding and catalytic activity [30, 31].

Somatic *EGFR* mutations serve as excellent examples to illustrate the mutational spectrum of RTKs. The entire EGFR TKD is encoded by exons 18–24. *EGFR* mutations predominantly cluster in exons 18–21, which are adjacent to the ATP-binding pocket [32]. Approximately 90% of these mutations are small in-frame deletions within exon 19 or L858R point mutation within exon 21 [33–35]. These mutations hyperactivate the kinase and, subsequently, its downstream signaling, conferring oncogenic properties [32, 36, 37]. Numerous large international clinical trials have now shown that patients whose tumors harbor activating somatic *EGFR* TKD mutations are uniquely sensitive to treatment with EGFR tyrosine kinase inhibitors (TKIs) [38–45].

Mutations can also occur in extracellular domain (ECD), transmembrane domain (TMD) and juxtamembrane domain (JMD) of RTKs. Three missense mutations within the EGFR ECD (P596L, G598 V, and A289V) were previously reported in glioblastoma (GBM) [46, 47]. These mutations are associated with increased expression of EGFR protein, which undergoes phosphorylation in the absence of ligand stimulation [46]. In contrast to lung cancer patients with *EGFR* TKD mutations, GBM patients with *EGFR* ECD mutations have shown disappointing clinical outcomes when treated with the EGFR TKIs, erlotinib and gefitinib [48, 49]. Studies suggest that the *EGFR* ECD mutations adopt the inactive conformation (compared to EGFR TKD mutations which adopt the active conformation), and the net effect is that EGFR ECD mutations may be better inhibited with EGFR targeted therapies that bind to the inactive form of the receptor [50]. Point mutations in the FGFR3 ECD (specifically, S249C) were reported in carcinomas of the uterine cervix [51]. These mutations result in unpaired cysteine residues, allowing abnormal receptor dimerization through intermolecular disulfide bonding [52]. Mutations within ECD of other RTKs have also been reported, including RET in thyroid cancer [53] and KIT in gastrointestinal stromal tumor (GIST) [54]. HER2 G660D and V659E mutations within the TMD act as driver mutations in non-small cell lung cancer (NSCLC) [55]. HER2 V659 mutations are also found in a patient with Li-Fraumeni syndrome [56]. These mutations disrupt specific protein-protein and protein-lipid interactions within the HER2 TMD that are essential for proper receptor dimerization [57]. It has been also shown that these two TMD mutations exhibit lower protein turnover than wild-type HER2 [58]. In in vitro models, HER2 V659E exhibits sensitivity to two TKIs - lapatinib [56] and afatinib [59], indicating TMD mutations could serve as actionable therapeutic targets. Finally, mutations within the JMD release autoinhibitory juxtamembrane interactions and subsequently hyperactivate these RTKs, such as KIT V560G and PDGFRA V561D mutation in GIST [54]. Therefore, mutations within the ECD, TMD and JM of RTKs adopt alternative activating mechanisms compared to mutations within the TKD. It has been observed that patients with GIST harboring mutations within the ECD, TMD, and/or JMD have different treatment response from TKD mutations to targeted therapy by using imatinib [54], a competitive inhibitor of KIT [60] and PDGFRA [61]. Gain-of-function mutations in the various subdomains of the RTKs described above are represented schematically in Fig. 1b.

### Overexpression and genomic amplification

Overexpression of RTKs has been found in a variety of human cancers: *EGFR* in GBM [62], lung [63], esophageal [64] and thyroid cancer [65]; *HER2*/*ErbB2* in lung [66], bladder [67], breast [68] and gastric cancer [69, 70]; and *MET* in lung [71] and gastric cancer [72]. Overexpression leads to increased local concentration of receptor, which results in elevated RTK signaling and overwhelms the antagonizing regulatory effects [73]. While gene amplification is the major mechanism which leads to overexpression of RTKs, additional mechanisms of RTK overexpression include transcriptional/translational enhancement [74, 75], oncogenic viruses [64], derailment of normal regulatory mechanisms such as loss of phosphatases [76] or other negative regulators [77, 78]. Regardless of mechanism, overexpression of RTKs has been associated with poor outcomes in some cancer patients, such as *EGFR* and *HER3* in breast cancer [79].

Gene amplification is characterized by a process that increases the copy number of a specific region of the genome [80]. Genomic amplification can occur as extrachromosomal elements (double minutes), repeated units at a single locus or distributed throughout the genome (distributed insertions) [81]. Double minutes tend to result in high level amplification (> 25 copies) while distributed insertions tend to low level amplification (5 to 25 copies) [62]. Gene amplification may be influenced by common chromosomal fragile sites, defects in DNA replication, or telomere dysfunction [80]. Amplification of many RTKs occurs in a variety of human cancers, such as *EGFR*, *ERBB2* and *MET* [80]. Other RTK amplifications have also been reported in human cancers, including *FGFR1* in lung and breast cancer [82, 83], *FGFR3* in breast and bladder cancer [84, 85], *ERBB4* in breast and gastric cancer [86, 87], *FLT3* in colon cancer [88], *KIT* in melanoma and GIST [89, 90], and *PDGFRA* in GBM [91]. Amplification patterns differ largely even in the same tumor type [62]. For example, a recent study in GBM indicated that 88% of cases with high-level *EGFR* genomic amplification showed EGFR protein overexpression by immunohistochemistry, in contrast to 36% of the cases with low-level *EGFR* amplification [62]. Lastly, RTK amplification can occur either in the context of a wild-type or mutated allele. For example, *EGFR* amplification was found to occur preferentially on the mutated allele in *EGFR*-mutant lung cancer [92]. RTK amplifications also act as an avenue for tumor cells to escape therapeutic treatment. For example, *MET* amplification and *HER2* amplification can be detected in *EGFR*-mutant lung cancers that become resistant to EGFR tyrosine kinase inhibitor therapy [93]. RTK overexpression is represented schematically in Fig. 1c.

### Chromosomal rearrangements

Genomic studies have identified numerous chromosomal rearrangements which lead to the formation of novel tyrosine kinase fusion oncoproteins [94–96]. The importance of identifying these chromosomal rearrangements and the ensuing tyrosine kinase fusion is underscored by that fact that these aberrant fusion proteins are often therapeutically targetable with small molecule inhibitors. The first tyrosine kinase fusion identified was BCR-ABL, which derived from translocation t(9,22) – the so called ‘Philadelphia Chromosome’ – which fuses the gene encoding the ABL1 tyrosine kinase on chromosome 9 to the *BCR* gene on chromosome 22, to form the BCR-ABL fusion oncoprotein [97]. BCR-ABL is characteristically found in patients with chronic myelogenous leukemia (CML) and in some patients with acute lymphoblastic leukemia [98, 99]. Notably, the first tyrosine kinase inhibitor developed and approved by the US Food and Drug Administration (FDA) – imatinib – targets the ABL kinase and has revolutionized the treatment of patients with CML [100, 101].

While BCR-ABL occurs exclusively in leukemia, many of the subsequently discovered tyrosine kinase fusions occur in multiple tumor types, including both liquid and solid malignancies. For example, the translocation t(2,5) fuses the gene encoding the ALK tyrosine kinase on chromosome 2 to the *NPM* gene on chromosome 5, to form the NPM-ALK fusion oncoprotein [102], which is found in approximately 50% of anaplastic large cell lymphoma (ALCL) [103]. Almost 30 years after the identification of the NPM-ALK fusion, similar ALK tyrosine kinase fusions have been found in other tumor types. Most notably, *ALK* rearrangements occur in approximately 3–7% of NSCLCs [104], approximately 50% of all inflammatory myofibroblastic tumors (IMTs) [105, 106], 10% of Spitzoid neoplasms [107], as well as small percentages in colon cancer [94, 108, 109], thyroid cancer [94, 110], and several other types of malignancies [94, 102, 111]. Likewise, oncogenic tyrosine kinase fusions involving *ROS1* have been identified in ~ 1% of NSCLCs [112], as well as in IMTs, cholangiocarcinoma, and GBM [94, 113]. RET kinase fusions have been recurrently detected in NSCLC and thyroid cancers [94, 114, 115]. Last but certainly not least, fusion oncoproteins involving the TRKA, TRKB, and TRKC tyrosine kinases (which are encoded by *NTRK1*, *NTRK2*, and *NTRK3*, respectively) have been identified across nine tumor types, including sarcoma, melanoma, gliomas, thyroid, lung, colon, breast, head and neck cancers) [94]. The fusion proteins have been reported as potent actionable targets in adult and children with TRK fusion positive cancers [116]. Numerous other tyrosine kinase fusions have been described, including those that incorporate EGFR [94, 117], HER2 [118], MET [94, 107], PDGFRa [119], and PDGFRb [94, 106]. These findings suggest that fusion events may have some common underlying etiology in human tumors. Several risk factors have been considered to contribute to the gene fusion events, including exposure to ionizing radiation [120, 121], topoisomerase poisons [122] and oxidative stress [123], but the precise molecular mechanisms remain elusive.

**Fig. 2:**
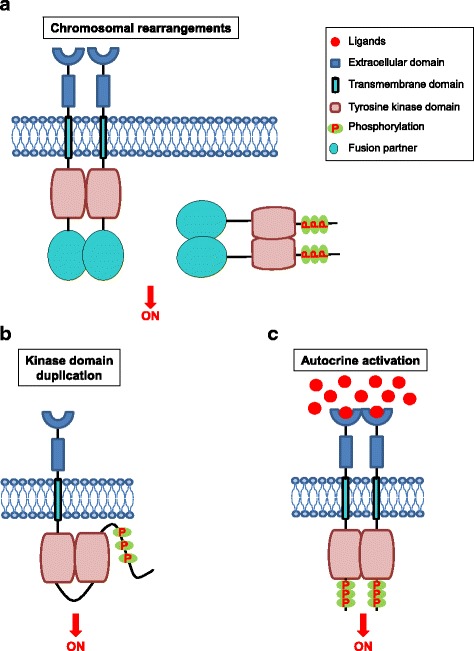
Mechanisms of oncogenic RTK activation. **a** Chromosomal rearrangements result in the formation of a hybrid fusion oncoprotein consisting partly of the RTK and partly of the fusion partner, a distinct protein (shown in the figure by the yellow oval). These RTK fusion proteins can be membrane bound (left side of the figure) or cytoplasmic (right side of the figure) depending on the location of the genomic breakpoint. In either case, the result is an activated kinase domain. **b** Duplication of the tyrosine kinase domain could possibly form an intra-molecular dimer in the absence of ligands, resulting in RTK activation. **c** Schematic representation of autocrine activation of RTK signaling. Increased local concentration of ligand activated the RTK, resulting in RTK dimerization, increased kinase activity, and phosphorylation of the receptor C-terminal tail

Despite the diversity of tyrosine kinase fusions which have been described, the structure of the resultant fusion oncoproteins retains a remarkable similarity. Fusions may occur in either the N-terminal or the C-terminal of the RTK, with the TKD preserved in both cases (Fig. 2a). If the genomic breakpoint occurs downstream of the exons encoding the full kinase domain (with preservation of the ECD, TMD, and JMD), then the resultant fusion protein will function as a membrane-bound receptor, such as the case for the EGFR-RAD51 fusion protein [117]. If the genomic breakpoint occurs upstream of the exons encoding the full kinase domain (with loss of the ECD, TMD, and JMD), then the resultant fusion protein will not be membrane bound. Instead, such proteins typically localize to the cytoplasm, as is the case for the EML4-ALK fusion protein [124]. Another characteristic of kinase fusions is the occurrence of multiple fusion partners within the same disease [94, 106, 125]. For example, there are at least nine known *ROS1* fusion partners found in NSCLC, including *SLC34A2*, *CD47*, *TPM3*, *SDC4*, *EZR*, *LRIG3*, *FIG*, *KDELR2* and *CCDC6* [94].

Although these partners can vary, they share three features. First, the regulatory unit of the fusion partner dictates the expression of the fusion, placing the tyrosine kinase oncoprotein under the endogenous promoter of the fusion partner [108, 126]. Second, most fusion partners contribute an oligomerization domain, which promotes ligand independent constitutive activation of the kinase [94, 127, 128]. The most common oligomerization domains found in the fusion partners are coiled-coil domains. For example, EML4-ALK, the most common ALK fusion detected in NSCLC, homodimerizes by virtue of a coiled-coil domain in EML4 [124]. Disruption of the coiled-coil domain abrogates the ability of EML4-ALK to transform cells [124]. Third, the fusion partner also determines subcellular localization of the fusion [129, 130], and this may have profound effects on the protein interactions that the fusion encounters, affecting activation, signaling, function, and degradation of the fusion. As such, RTK fusions can regulate similar cell signaling pathways as the ‘parental’ RTK from which they are derived (including RAS/MAPK, PI-3 K/AKT, and JAK2/STAT [106, 117]) and/or possibly even new pathways based on their altered cellular localization.

Chromosomal rearrangements of RTKs lead to chimeric fusion proteins, which contribute to oncogene addiction [106, 117]. Inhibiting RTK fusions with target specific TKIs has proven to be an effective therapeutic strategy across numerous types of RTK fusion driven cancers – including ALK in ALCL [131], IMT [132] and lung cancer [133], RET in lung and thyroid cancer [134–137], ROS1 in GBM [138], lung cancer [139], and IMT [106], EGFR in lung cancer [117], and NTRK in IMT [140], lung [141], kidney [141], colon [140, 141] and other types of cancer [141].

#### Constitutive activation by kinase domain duplication

Intragenic partial duplication is a type of chromosomal rearrangement that confers cancer cells the ability to acquire new protein isoforms [142]. Kinase domain duplications (KDDs) constitute one type of intragenic partial duplication, resulting in a novel mechanism for RTK activation in tumor cells. For example, oncogenic *EGFR*-KDD and *BRAF*-KDD have been reported in human cancers, along with their responses to the respective targeted therapies against EGFR and BRAF. Recently, our group reported that *EGFR*-KDD is recurrently found in NSCLC [143]. We also found that *EGFR*-KDD occurred in other types of human tumors, including gliomas, sarcoma and Wilms’ tumor [143]. *BRAF*-KDD has been reported in gliomas and advanced acinic cell tumor [144, 145]. BRAF is an intracellular serine/threonine kinase; however, we discuss here as demonstration of principle. Most recently, a group of investigators has analyzed clinical genomic data from 114,200 human tumors and found recurrent KDD alterations involving several kinases, including the ErbB family (*EGFR*, *ERBB2* and *ERBB4*), FGFR family (*FGFR1, FGFR2* and *FGFR3*), NTKR family (*NTRK1* and *NTRK2*), PDGFR family (*PDGFRA* and *PDGFRB*), and other kinases (*BRAF, RET, MET, ROS1, ALK* and *KIT*) [146]. In brain tumors, KDD occurs most frequently within *EGFR*, *BRAF*, *PDGFRA*, and *FGFR3*. In extracranial tumors, KDD was frequently found in *RET*, *MET* and *ALK* genes [146]. Overall, the frequency of KDD alterations was 0.62% (598 total KDDs in 114,200 cases analyzed).

In nature, gene duplication is one method by which species introduce genetic novelty or redundancy, thereby allowing them to adapt to various environmental conditions [147]. It is possible that KDDs in tumor cells can be selected for in response to pressure exerted by cancer therapy. For example, *BRAF*-KDD was identified as a new mechanism of drug resistance in patients with melanoma after BRAF inhibitor treatment [142]. Identification of EGFR-KDD amplification in the post-treatment biopsy suggested that KDD is also involved in the acquired resistance of EGFR TKI, afatinib [143].

To date, the most well studied KDD is the EGFR-KDD [143]. In normal biology, the presence of EGF ligands activates wild-type EGF receptor through the formation of an asymmetric dimer between two receptor molecules [9]. Considering that EGFR-KDD contains two tandem, in-frame tyrosine kinase domains, it is possible that the mode of activation of the EGFR-KDD variant involves constitutive intra-molecular dimerization (Fig. 2b). Therefore, for this variant, EGFR signaling can be activated in a ligand independent manner. Preclinical modeling of the *EGFR*-KDD protein validated this potential activation mechanism in silico and in vitro. Notably, EGFR-KDD activation is quite distinct from the molecular mechanisms governing activation of EGFR kinase domain mutants described above (e.g., L858R, exon 19 deletion), underscoring the importance of considering how genomic findings alter protein structure and function to result in an oncogenic variant.

With respect to BRAF-KDD, most of the genomic breakpoints occur in intron 9 of *BRAF*, which generates a truncated protein that dimerize in a RAS-independent manner [148]. Thus, BRAF-KDD adopts a complete different activating mechanism from EGFR-KDD, which give us important clues that possibly KDD in different RTKs use different activation mechanisms. Systematic functional studies of each of the novel identified KDD within RTK are very necessary for the understanding of the entire RTK paradigm.

### Autocrine activation

Cell-cell communication utilizes “messengers” – such as growth factors and cytokines – that are released by secretory cells and delivered to remote target cells. “Autocrine” refers to the situation that the target cells are secreting cells themselves [149]. Constitutive autocrine activation might lead to clonal expansion and tumor formation (Fig. 2c) [150], and autocrine activation of various RTKs has been well characterized in diverse cancers, including TGFα-EGFR [151], HGF-MET [152, 153], and SCF-KIT autocrine loops [154–156]. RTK autocrine loop may work synergistically with other autocrine growth pathway and drive tumor development. The growth advantage conferred by SCF-KIT loop partially synergizes with another two autocrine loops, IGF-l and bombesin, to drive the development of small cell lung cancer (SCLC) [154]. Autocrine pathways could act as a rational target for cancer therapy [151]. For example, ligand/receptor autocrine loops renders EGFR-mutant lung cancer cells less sensitive to EGFR TKI inhibition [157].

## Emerging mechanisms to aberrantly activate RTKs

### MicroRNAs

MicroRNAs can directly modulate the expression of RTKs, and function as both tumor suppressors and oncogenes [158]. For example, microRNA-10a promotes metastasis by directly regulating EPH4A-mediated epithelial-mesenchymal transition and adhesion in hepatocellular carcinoma [159]. MicroRNA-145 suppresses the development of lung adenocarcinoma through directly modulating *EGFR* expressions at both mRNA and protein levels [160]. MicroRNA-219-5p suppresses GBM development through repressing *EGFR* expression by directly binding to its 3’-UTR [161]. In addition, microRNAs have also been shown to be involved in the RTK signaling and regulation of tumor formation. Recent data has demonstrated that RTKs, such as MET, EGFR, and PDGFR, regulate microRNA-134 in GBM, while microRNA-134 acts as a tumor-suppressive hub and controls KRAS and STAT5B expression levels [162]. Insights into oncogenic microRNAs and RTK signaling will allow exploiting and improving cancer therapies. For example, the combination of a monoclonal antibody against EGFR and an inhibitor of microRNA-21 improve the treatment outcome in GBM [163]. Moreover, microRNAs could function as potential prognostic markers and assist in patient stratification. The microRNA signature (MiR-99a/Let-7c/miR-125b) may serve as biomarker for prognosis of patients with colorectal cancer treated with anti-EGFR antibodies [164]. An improved understanding of microRNAs involved in RTK signaling may have future implications in cancer detection, therapy and prognosis.

### Alterations in tumor microenvironment

Several notable advances have been made during the last decade in the recognition of the importance of tumor microenvironment, especially tumor vasculature and tumor stroma [165]. Members of the Eph receptor family mediate cell-cell interaction in tumor stroma and tumor vasculature [166]. Macrophages function as key cellular components of tumor microenvironment. *AXL* is highly expressed within tumor associated macrophages where AXL may promote immunosuppressive and pre-neoplasia phenotypes [167]. RET and GFRA1 have been shown to be expressed in stromal cells of the bone marrow microenvironment and implicated in the development of acute myeloid leukemias [168]. Many other RTKs have been shown to be important in the tumor microenvironment, including VEGFR [169, 170] and PDGFR [171]. As such, these RTKs represent attractive potential targets for drug design. Many AXL inhibitors have been detected and are efficacious in preclinical studies against cancer [167].

### Signal attenuation by negative regulators

The activity of RTKs must be tightly regulated and properly balanced in order to mediate their normal cellular activities and physiological processes. Signal attenuation and downregulation of RTK pathways provide important implications in cancer therapeutics and several well characterized negative regulators in RTK signaling (such as *PTEN*, *LRIG1* and *ERRFI1*) are bona fide tumor suppressors [172–174].

*ERRFI1* (ErbB Receptor Feedback Inhibitor 1) *–* which encodes the protein MIG6 – is located within chromosome 1p36.1–3, a hotspot region frequently deleted in a broad range of human cancers, including breast, liver and kidney cancers [175]. MIG6 has been described to be mutated in different human cancers [176, 177]. MIG6 expression is also downregulated or silenced in skin, breast, pancreatic and ovarian carcinomas [178, 179]. Loss of *Errfi1* in mice leads to abnormal activation of EGFR signaling and is associated with a high incidence of neoplastic lesions [178]. These findings suggested that MIG6 played tumor suppressive roles possibly involved in EGFR signaling. MIG6 contains two functional regions, termed segments 1 and 2 which are 77 amino acids in total [174]. Structural studies indicate that MIG6 (segment 1) is able to inhibit EGFR kinase activity in the presence of the asymmetric dimer. MIG6 (segment 1) binds to ‘activator’ kinase and prevents the activation of EGFR, while segment 2 is required for the inhibition of the kinase activity of activated EGFR, and that both segments 1 and 2 are essential for the potent inhibition of EGFR activity [174]. Residues in the binding interface between EGFR and MIG6 (segment 1) are conserved across all ErbB family members rather than other protein kinases [9], However, in another structural study, MIG6 could not effectively inhibit the oncogenic mutants of EGFR (e.g. L858R), presumably because EGFR mutants can form asymmetric dimers at a lower energetic cost than wild-type EGFR [36]. The C-lobe is less accessible by MIG6 in configurations that more strongly favor formation of asymmetric dimers [32]. These two studies give us clues that MIG6 may potentially inhibit EGFR-KDD, EGFR-RAD51 and EGFR-PURB, because these EGFR mutant proteins have intact wild-type TKD which could potentially act as ‘activator’ kinase in the form of activating asymmetric dimerization.

## RTKs as therapeutic targets

Since RTKs play crucial roles in cancer development, targeting oncogenic driver mutations of RTKs has revolutionized the treatment of cancer patients. Above, we touched on how targeted therapies are deployed in specific clinical scenarios for patients whose tumors harbor oncogenic RTK variants. However, a detailed review of all RTK inhibitors in the treatment of human tumors is beyond the scope of this manuscript. In brief, many small-molecule inhibitors have been developed for treating cancers and other diseases caused by driver mutations within RTKs. These inhibitors specifically target the ATP-binding site of the intracellular TKD [180]. In addition, the US FDA has approved many monoclonal antibodies that interfere with RTK activation, including cetuximab in lung cancer [181], panitumumab in colon cancer [182], cetuximab in head and neck cancer [183], trastuzumab and pertuzumab in breast cancer [184, 185]. Overall, the development and routine clinical implementation of agents (TKIs and monoclonal antibodies) targeting RTKs has heralded the new age of precision cancer medicine. Despite these advancements, acquired resistance to targeted therapies inevitably develops [40, 133]. Acquired resistance can occur through either acquired genomic alterations [186, 187] or activation of critical signaling pathways [188–190]. Novel approaches have been shown to effectively overcome acquired resistance, including the development of second-generation [191, 192] and third-generation inhibitors [193, 194] and the combinational use of TKIs with monoclonal antibodies against the same target [195].

## Conclusions

Our understanding of RTK signaling has advanced dramatically in the past two decades. Studies of RTKs have provided fundamental insight into how this protein family functions and how to develop targeted therapeutics. However, much work is still required to fully understand all members of the RTK family. An improved understanding of RTK signaling pathways will provide a strong foundation on which improvements to patient care can be made. An integrated approach, combining genetic, cellular, biochemical, and structural modeling techniques, may offer the most complete view yet of this critical family of protein tyrosine kinases.

## Abbreviations

ALCL: Anaplastic large cell lymphoma;
CML: Chronic myelogenous leukemia
ECD: Extracellular domain
FDA: Food and Drug Administration
GBM: Glioblastoma
GIST: Gastrointestinal stromal tumor
IMT: Inflammatory myofibroblastic tumor
IR: Insulin receptor
JMD: Juxtamembrane domain
KDD: Kinase domain duplication
NGS: Next generation sequencing
NSCLC: Non-small cell lung cancer
PTB: Phosphotyrosine-binding domain
RTK: Receptor tyrosine kinases
SCLC: Small cell lung cancer
SH2: Src homology-2 domain
TKD: Tyrosine kinase domain
TKI: Tyrosine kinase inhibitor
TMD: Transmembrane domain
